# Digital Removable Partial Denture Fabrication Using an Intraoral Scanner and the Altered-Cast Technique for a Patient With Free-End Partial Edentulism: A Case Report

**DOI:** 10.1155/crid/3588047

**Published:** 2025-11-12

**Authors:** Yuta Iida, Yukio Kameda, Akinori Tasaka, Shuichiro Yamashita

**Affiliations:** ^1^Chugoku/Shikoku Branch, Japan Prosthodontic Society, Okayama, Japan; ^2^Higashi-Kanto Branch, Japan Prosthodontic Society, Saitama, Japan; ^3^Department of Removable Partial Prosthodontics, Tokyo Dental College, Tokyo, Japan

**Keywords:** altered-cast technique, CAD/CAM, case report, intraoral scanner, removable partial denture

## Abstract

This clinical report describes treatment with a removable partial denture produced using an anatomical impression made with an intraoral scanner and a mucocompressive impression made using the altered-cast technique. First, digital scanning was performed with an intraoral scanner to obtain an anatomical impression of the remaining teeth and the residual ridges. Computer-aided design software was then used to design the framework, which was fabricated by selective laser melting. A mucocompressive impression was subsequently made using the altered-cast technique, and gypsum was poured to modify the cast accordingly. The denture was then fabricated. The patient was satisfied with the stability, retention, and overall function of the prosthesis.

## 1. Introduction

Digital technology in prosthetic dental treatment has advanced dramatically in recent years. A computer-aided design and manufacturing (CAD/CAM) workflow using data obtained from an intraoral scanner (IOS) is now commonly used for the production of fixed prostheses, such as crowns and bridges and implant superstructures [1]. In addition, several companies have introduced systems for producing complete dentures by scanning impressions with dental laboratory scanners and fabricating artificial teeth and denture bases via milling or three-dimensional (3D) printing [2]. Furthermore, removable partial dentures (RPDs) can now be produced by designing the framework using CAD software and fabricating it by metal additive manufacturing (selective laser melting [SLM]). Satisfactory results have been reported with the use of an IOS and CAD/CAM technology to create tooth-supported RPDs [3, 4]. In addition, the application of digital techniques to the design and fabrication of combined tooth–implant-supported RPDs has also shown promising clinical outcomes [5]. Several clinical reports have demonstrated the use of intraoral scanning in the fabrication of tooth–tissue-supported RPDs [6]. However, the application of IOS in tooth–tissue-supported RPDs presents unique challenges due to the differing physical characteristics and displaceability of teeth and mucosa. Although teeth are generally more stable than the residual ridge mucosa, they also exhibit slight movement under functional pressure. These differences require separate impression approaches, which cannot be fully captured by digital methods alone. Few studies have provided detailed information regarding the clinical challenges of mucosal displacement following digital impressions or long-term follow-up outcomes. When taking impressions for tooth–tissue-supported RPDs, the remaining teeth and the residual ridge mucosa exhibit different amounts of displacement under pressure and must therefore be considered separately [7, 8]. An anatomical impression of the remaining teeth must be taken, and an IOS, which does not exert any pressure while taking impressions, provides an accurate record of the shapes of these teeth and their positional relationships in the dentition. An IOS cannot be used to take a functional impression because it does not apply pressure to the mucosa of the residual ridge. The altered-cast technique is a method of using a framework to take a pressure impression of the residual ridge [9], and Suzuki et al. described a technique for creating tooth–tissue-supported RPDs for partially edentulous patients by combining this method with the use of an IOS [10]. While their report demonstrated clinical usefulness, it lacked detailed discussion of procedural limitations or long-term clinical outcomes, leaving open questions regarding the broader applicability of this approach. In contrast, the present case report provides a detailed account of the clinical challenges and considerations involved in applying the altered-cast technique following digital impression acquisition. Furthermore, a 4-year clinical follow-up is presented, offering not only evidence of prosthesis longevity and stability but also practical validation of this hybrid digital–conventional workflow in a real-world setting. This approach has the potential to improve patient comfort by reducing invasive impression procedures, increase efficiency through reduced chairside time, and enhance the reproducibility of prosthetic components. Although this is a single case report, the techniques described may be applicable to similar clinical situations, supporting the future integration of digital workflows into the management of partially edentulous patients.

In this paper, a case in which an RPD was produced using an anatomical impression taken with an IOS and a pressure impression taken using the altered-cast technique, and satisfactory results were obtained over a 4-year period, is reported.

## 2. Case Report

**Figure 1:**
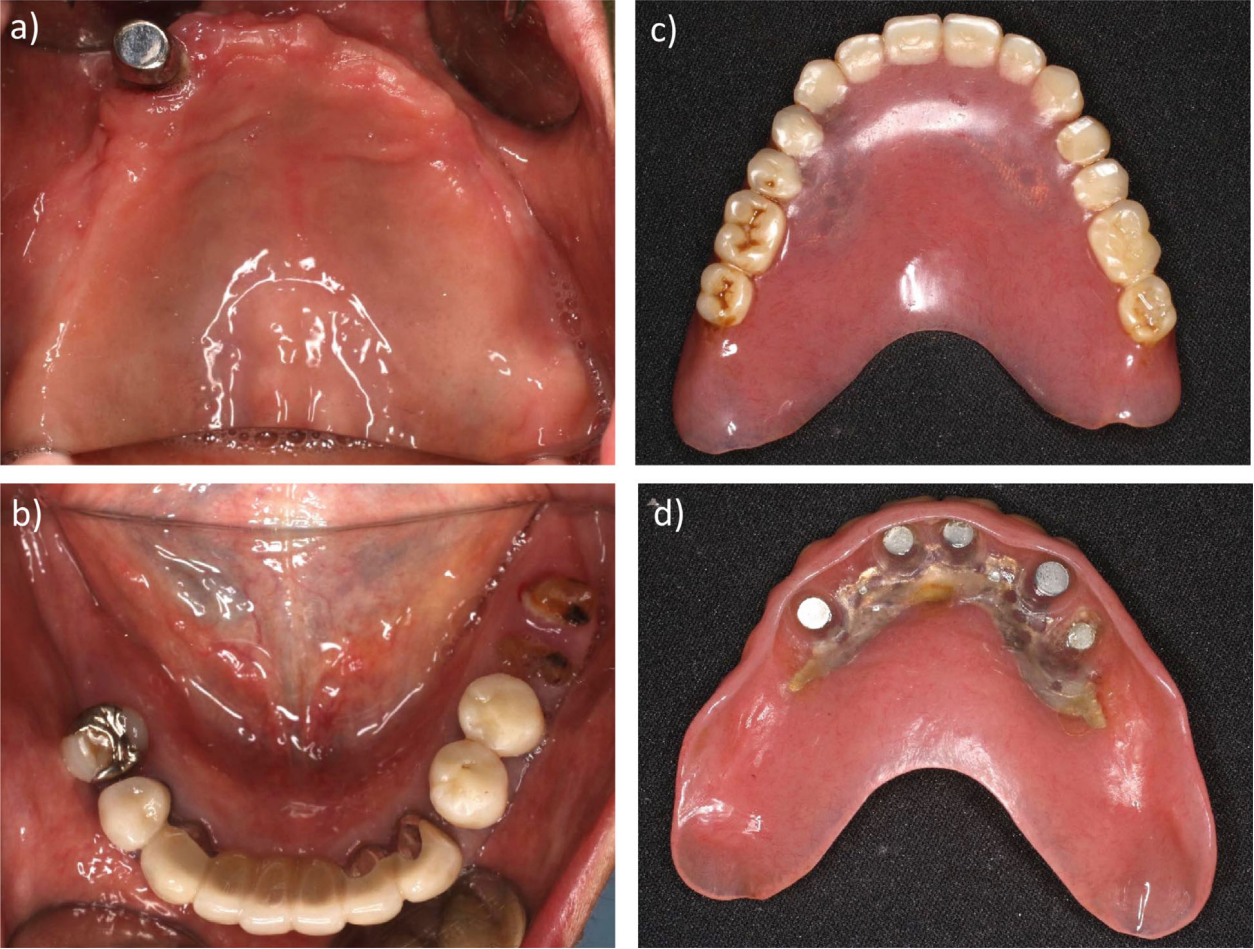
Intraoral photographs and photographs of dentures on first examination. (a) Maxillary occlusal view. (b) Mandibular occlusal view. (c) Occlusal surface of maxillary denture. (d) Tissue surface of maxillary denture.

**Figure 2:**
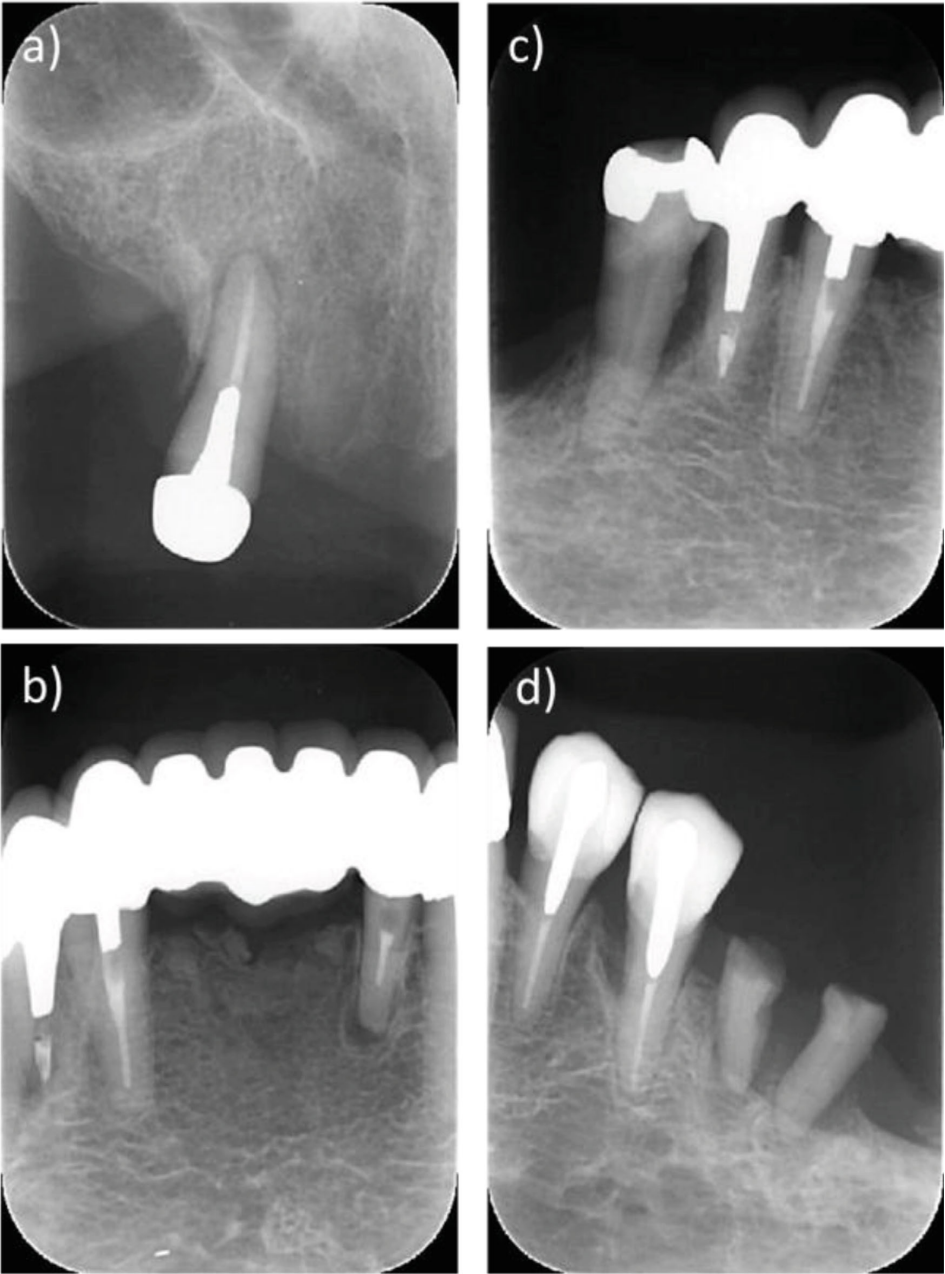
X-rays on first examination. (a) Tooth #13 (maxillary right canine). (b) Anterior mandibular teeth. (c) Mandibular right teeth. (d) Mandibular left teeth.

A 79-year-old woman presented with poor retention for a maxillary overdenture and missing mandibular molars (#37, #46, and #47: FDI two-digit system) (Figure 1). The maxillary overdenture, retained by a magnetic attachment at #13, was ill-fitting and lacked retention. Several teeth (#36 residual root, #13 broken root, and #45 affected by severe periodontitis) were deemed nonrestorable (Figure 2). Before treatment, her summary score on the Japanese version of the Oral Health Impact Profile (OHIP-J; Yamazaki 2007, 10.1111/j.1365-2842.2006.01693.x.) was 153.

The necessity of refabricating the maxillary denture and of prosthetic treatment for the mandibular missing teeth was explained to the patient. Two treatment plans were prepared for the mandibular jaw, one using implants and the other with an RPD, and informed consent for the latter was obtained. The patient had previously had bad experiences during the removal of mandibular jaw impressions, and it was therefore suggested that digital technology using an IOS be used to create the RPD, to which she consented.

A vertical bone defect was evident at #35, but after initial periodontal therapy, a long epithelial attachment was achieved, and the periodontal pocket improved from 7 to 3 mm. As a result of improved stress resistance, the #34 crown was connected to the #35 crown. Treatment dentures were fitted to the maxillary and mandibular jaws, and after confirming that stable mastication was possible, final prosthetic treatment was performed. A guiding plane and rest seat were prepared on the abutment tooth #44, and metal-bonded crowns for #34 and #35 were fabricated to match the path of insertion and removal. The maxillary complete denture was fabricated concurrently using the conventional method, whereas the mandibular RPD was digitally designed and fabricated.

**Figure 3:**
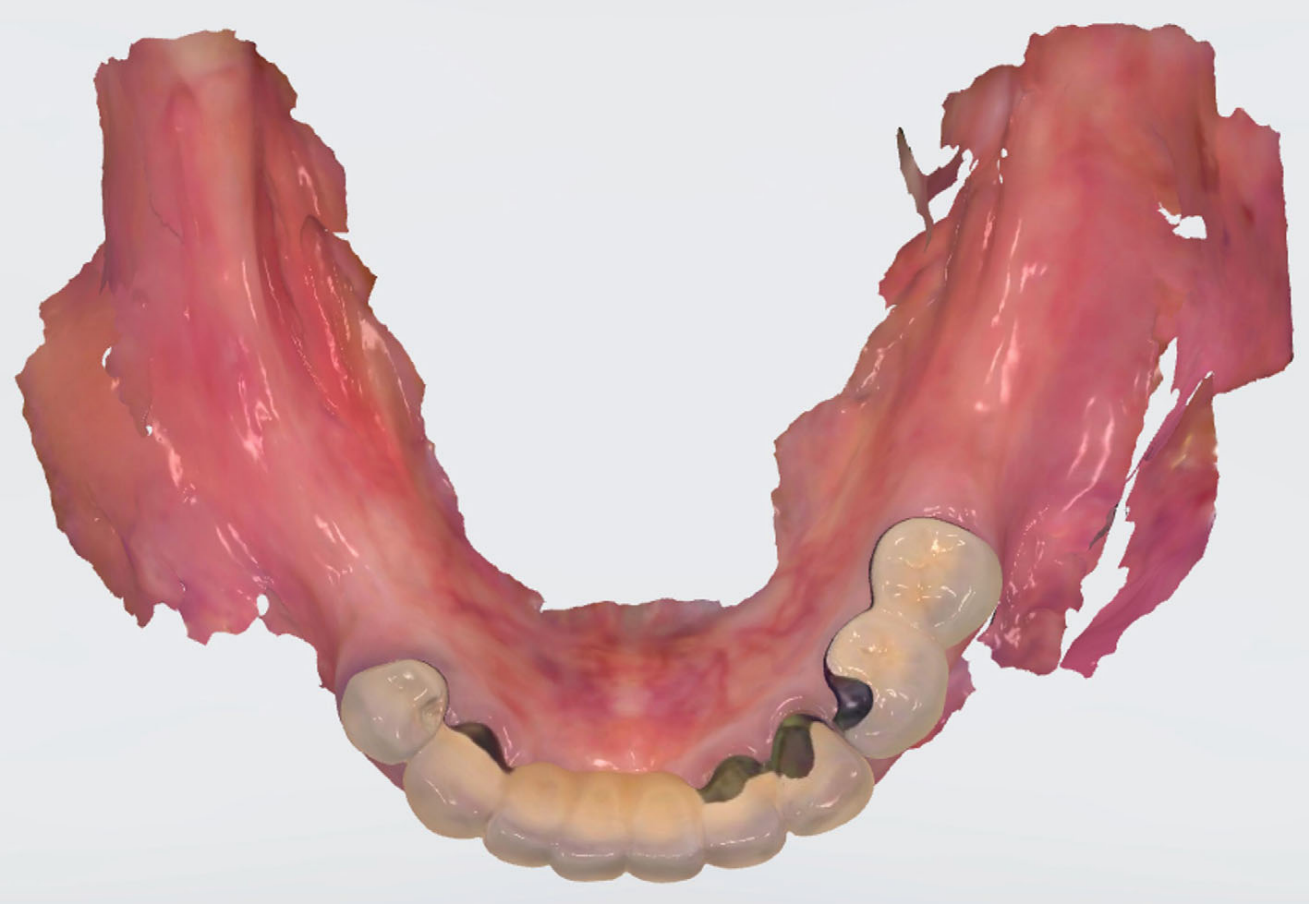
IOS scanning data.

**Figure 4:**
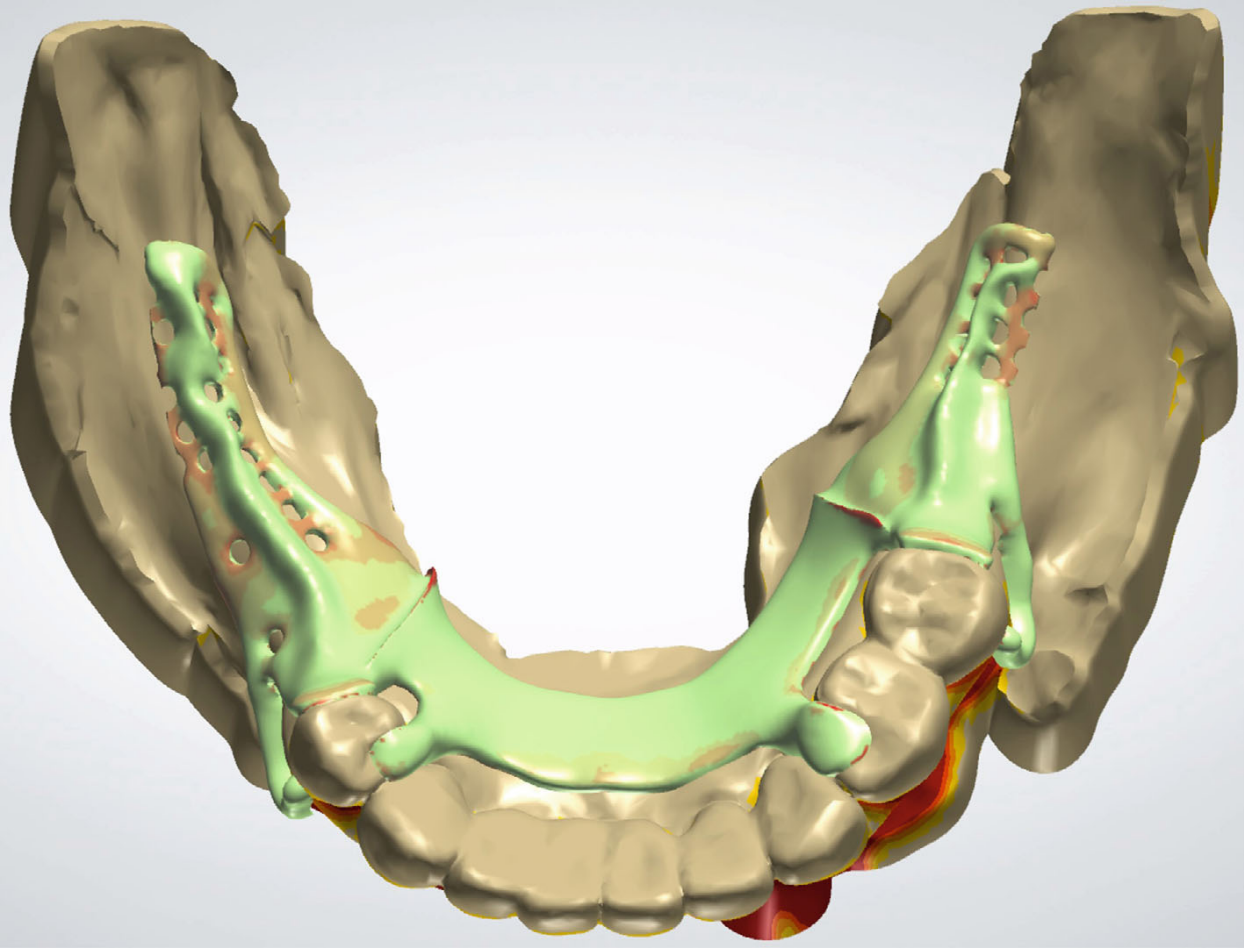
RPD framework design.

**Figure 5:**
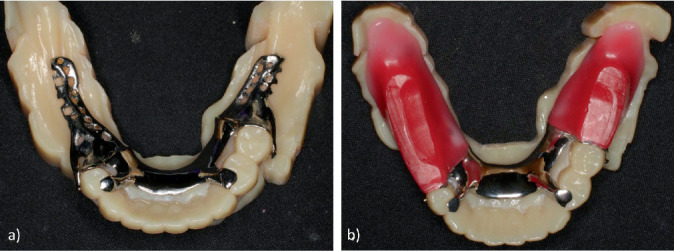
RPD framework. (a) SLM framework on the 3D model. (b) SLM framework with the bite plate attached.

**Figure 6:**
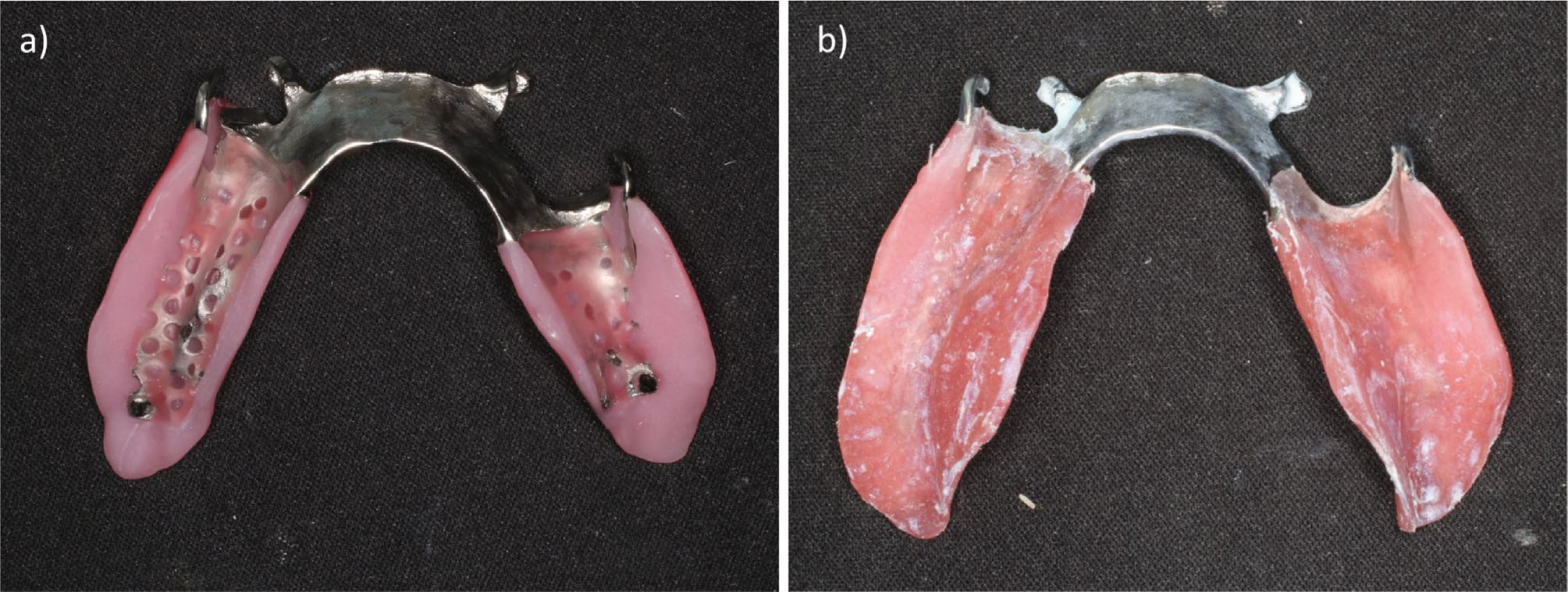
Framework with the bite plate attached before use of the altered-cast technique. (a) Uncorrected outline of the bite plate. (b) Corrected outline of the bite plate.

**Figure 7:**
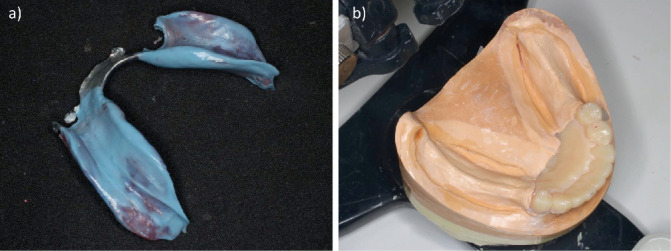
Altered-cast technique. (a) The mucosal surface of the bite plate after the use of the altered-cast technique. (b) 3D model of the residual ridge reconstructed with gypsum.

**Figure 8:**
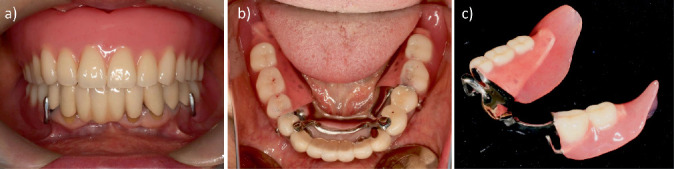
Intraoral photographs and photograph of dentures after treatment. (a) Frontal view. (b) Occlusal view. (c) Mandibular denture.

First, digital scanning was conducted with an IOS (Trios 3, 3Shape, Copenhagen, Denmark) to obtain an anatomical impression of the remaining teeth and the residual ridge (Figure 3). The scanning procedure covered the mandibular arch including the residual ridge and abutment teeth, following the manufacturer's recommended protocol from posterior to anterior regions. Multiple scans were performed as needed to ensure accuracy. CAD software (Dental System; 3Shape) was then used to design the framework. The design included rests on #34 and #44, I-bar clasps on #35 and #44 for the direct retainer, and a lingual bar as the major connector (Figure 4). The framework was then fabricated as a cobalt–chrome alloy using metal laser sintering equipment (EOSINT M270, EOS, Krailling, Germany). A 3D printer (EDEN 260S, Stratasys, Eden Prairie, MN, United States) was also used to produce a 3D model, and the framework was fitted to this model. Next, the base plates were attached to the retention grid of the framework on the model, and the occlusal rim was constructed on top of them to produce the bite plate (Figure 5). The fit of the framework inside the mouth was then checked, and maxillomandibular registration was recorded at the same time as occlusal interference was adjusted. After this, the shape of the borders of the bite plate was corrected using autopolymerizing resin (Unifast III, GC, Tokyo, Japan), and a chemically cured hard denture relining material (Tokuyama Rebase III Fast, Tokuyama Dental, Tokyo, Japan) was used to improve the fit on the mucosal surface (Figure 6). After bite plate correction, a pressure impression of the alveolar ridge mucosa was taken by the altered-cast technique using silicone rubber impression material (Exadenture, GC). While this impression material was hardening, it was held in place by manual pressure while checking that the rest fit properly into the rest seat. Having confirmed that the shape of the mucosal surface was reproduced in the impression, silicone bite registration material (Exabite III, GC) was used to record the final maxillomandibular registration. The alveolar ridge of the 3D model was removed, and after checking that the framework was reinstated to its proper position, the model was reconstructed using Type 4 dental stone (New Fujirock, GC) (Figure 7). The artificial teeth were then arranged, and trial fitting of a wax denture was carried out, before finalizing the denture with heat-curing resin (Proimpact, GC) (Figure 8).

**Figure 9:**
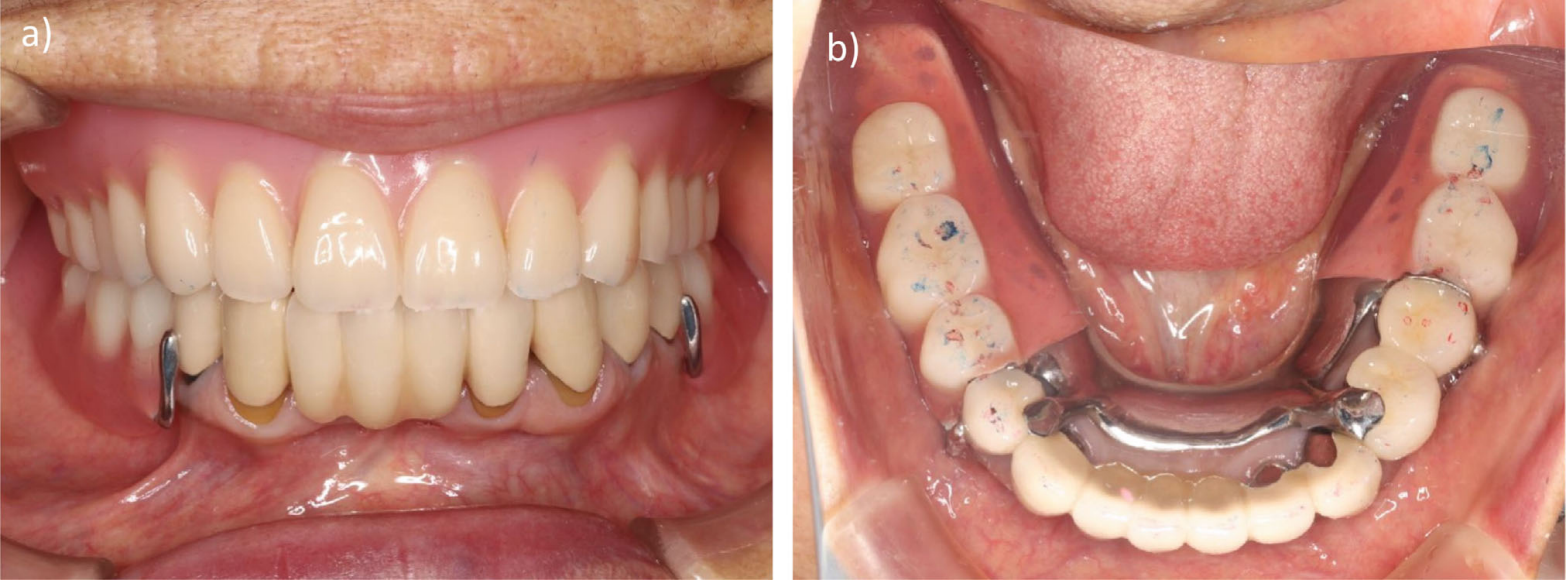
Intraoral photographs 4 years after treatment. (a) Frontal view. (b) Occlusal view.

One month after denture delivery, the patient was comfortable and satisfied with the stability, retention, and overall function of the RPD. Her OHIP-J summary score after the procedure was 113, an improvement over her score before the procedure. She has now been wearing the dentures for 4 years, and the fit and occlusal contacts are being checked at recall appointments every 3 months, but no issues have been noted (Figure 9).

## 3. Discussion

To produce the framework in the present case, it was first designed using the digital scanning data obtained from the IOS and then fabricated by SLM. According to a systematic review, SLM offers better trueness and mechanical strength than frameworks fabricated using the casting technique [11]. The framework fabricated for the present patient provided a satisfactory fit both to the 3D model and inside the mouth. Regarding the periodontal condition and mobility status of the abutments, regular examinations over the 4-year follow-up period showed stable periodontal health, with no significant increase in pocket depth, no bleeding on probing, and no abnormal tooth mobility observed. Although indirect retainers were omitted in this case due to esthetic and spatial limitations, this design should be regarded as a clinical compromise rather than a recommended standard. The altered-cast technique and extended denture base design contributed to stable support. Consequently, no clinical problems were observed, and relining was not required during the 4 years of follow-up.

The altered-cast technique was first proposed by Applegate [9], and it involves producing a metal framework and a tray for the alveolar ridge on the gypsum model obtained by anatomical impression to create a pressure impression of the mucosal surface. In the present case, 3D models were produced from the IOS data, which reproduced the shape of the alveolar ridge in a manner corresponding to the anatomical impression. The accuracy of intraoral scanning of partially edentulous dentition reportedly decreases at the residual ridge compared with the teeth [12]. The IOS stitches together scanned 3D images to complete the final digital 3D dataset, and errors may be more likely to occur in flatter areas with fewer irregularities than the remaining teeth, such as the residual ridge [13]. A recent in vitro study also demonstrated that the accuracy of intraoral scanning is lower in the edentulous maxilla's peripheral zones, including the palatal vault and vestibular regions, compared with central areas, due to limited surface features and scan accessibility [14]. It is difficult to capture the functional morphology of the oral vestibule, lips, tongue, cheeks, and other mobile tissues with an IOS [15]. In the present case, it was not possible to scan the retromolar pad, mylohyoid line, or other necessary landmarks. The denture base outline was thus smaller than the ideal shape required for a free-end saddle partial denture, and the outline was therefore corrected inside the mouth with autopolymerizing resin (Figure 6). The mandibular accuracy of IOS scanning of the residual ridge also meant that the mucosal surface of the bite plate did not fit well, and denture reline was used to correct the fit of the mucosal surface before using the altered-cast technique. For free-end saddle partial dentures, the prosthesis will only function properly if an anatomical impression of the remaining teeth and pressure impression of the residual ridge mucosa are taken. It was previously impossible to take a complete anatomical impression without applying any pressure at all, but the use of an IOS has made it feasible to conduct scanning inside the mouth with no pressure, and the results in the present case suggest that this method of combining IOS with the altered-cast technique may be a reasonable technique for appropriately correcting the amounts of displacement under pressure of the remaining teeth and the residual ridge mucosa. Though this technique offers clinical advantages, it is important to note that its reproducibility depends on case selection, operator experience, and laboratory collaboration. In addition, the initial cost and technical training required for mastering the hybrid workflow may limit its immediate adoption in all practice settings. Several digital methods have been proposed to replicate the altered-cast technique using general-purpose CAD software, which allows for complete virtual modifications of the residual ridge portion of the model. However, these methods often require advanced digital design skills and may not be easily reproducible in daily clinical practice. In contrast, the present technique uses intraoral scanning combined with conventional chairside relining and the altered-cast procedure, offering a more accessible alternative. Furthermore, the good 4-year clinical outcome supports its feasibility and long-term effectiveness in real-world settings. Several methods using digital technology for the altered-cast technique have been reported; however, they often require the use of general-purpose CAD software, which necessitates operators with advanced CAD skills. Therefore, further development of dental-specific CAD software is needed to streamline the clinical application of these techniques [16–18].

## 4. Conclusion

In the present case, an RPD for mandibular free-end partial edentulism was fabricated by combining an anatomical impression taken with an IOS and a pressure impression taken using the altered-cast technique, and satisfactory results were obtained by appropriately correcting the amounts of displacement under pressure of the remaining teeth and the residual ridge mucosa.

This combined technique offers advantages to patients by providing improved fit, comfort, and function of the prosthesis while minimizing the need for adjustments. For clinicians, it facilitates more accurate impression-making with digital technology, streamlines the fabrication process, and enhances long-term clinical outcomes.

## Data Availability

The data that support the findings of this study are available from the corresponding author upon reasonable request.
